# Comparison of the efficacy of seven non-surgical methods combined with mechanical debridement in peri-implantitis and peri-implant mucositis: A network meta-analysis

**DOI:** 10.1371/journal.pone.0305342

**Published:** 2024-08-14

**Authors:** Yingjie Bai, Shengao Qin, Bingshuai Lu, Weiyi Wang, Guowu Ma

**Affiliations:** 1 School of Stomatology, Dalian Medical University, Dalian, China; 2 Academician Laboratory of Immune and Oral Development & Regeneration, Dalian Medical University, Dalian, China; 3 Salivary Gland Disease Center and Beijing Key Laboratory of Tooth Regeneration and Function Reconstruction, Beijing Laboratory of Oral Health and Beijing Stomatological Hospital, Capital Medical University, Beijing, China; 4 Beijing Laboratory of Oral Health, Capital Medical University, Beijing, China; 5 School of Stomatology, Hainan Medical University, Haikou, China; 6 Department of Stomatology, Stomatological Hospital Affiliated School of Stomatology of Dalian Medical University, Shahekou District, Dalian, China; Klinikum der Johann Wolfgang Goethe-Universitat Frankfurt, GERMANY

## Abstract

This network meta-analysis aims to compare the clinical efficacy of seven non-surgical therapies for peri-implant disease, including laser treatment, photobiomodulation therapy (PBMT), photodynamic therapy (PDT), systemic antibiotics (SA), probiotics, local antimicrobials (LA), and air-powder polishing (APP) combined with mechanical debridement (MD). We conducted searches in four electronic databases, namely PubMed, Embase, Web of Science, and The Cochrane Library, to identify randomized controlled trials of non-surgical treatments combined with MD for individuals (aged at least 18 years) diagnosed with peri-implantitis or peri-implant mucositis with a minimum of 3 months follow-up. The outcomes of the study were the reduction in pocket probing depth (PPD) and bleeding on probing (BoP), plaque index (PLI), clinical attachment level (CAL), and marginal bone loss (MBL). We employed a frequency random effects network meta-analysis model to combine the effect sizes of the trials using standardized mean difference (SMD) and 95% confidence intervals (CIs). Network meta-analyses include network plots, paired comparison forest plots, league tables, funnel plots, surface under the cumulative ranking area (SUCRA) plots, and sensitivity analysis plots. The results showed that, for peri-implantitis, PBMT +MD demonstrated the highest effect in improving PPD (SUCRA = 75.3%), SA +MD showed the highest effect in improving CAL (SUCRA = 87.4%, SMD = 2.20, and 95% CI: 0.38 to 4.02) and MBL (SUCRA = 99.9%, SMD = 3.92, and 95% CI. 2.90 to 4.93), compared to MD alone. For peri-implant mucositis, probiotics +MD demonstrated the highest effect in improving PPD (SUCRA = 100%) and PLI (SUCRA = 83.2%), SA +MD showed the highest effect in improving BoP (SUCRA = 88.1%, SMD = 0.77, and 95% CI: 0.27 to 1.28), compared to MD alone. Despite the ranking established by our study in the treatment of peri-implant disease, decisions should still be made with reference to the latest treatment guidelines. There is still a need for more high-quality studies to provide conclusive evidence and especially a need for studies regarding direct comparisons between multiple treatment options.

## Introduction

Periodontal disease, dental caries, and trauma can lead to tooth loss, which seriously affects people’s oral health. Currently, oral implant surgery is the first choice for replacing missing teeth, which is done by placing implants into the alveolar bone. In comparison to other restorative options like removable partial dentures or fixed bridges, dental implant offers several advantages, including the absence of damage to neighboring teeth, excellent retention and support, comfort. However, factors such as periodontal disease, poor oral hygiene, smoking, and systemic diseases increase the risk of peri-implant disease. Peri-implant disease is a common biological complication encountered in clinical practice. It can be defined as the occurrence of inflammatory damage in the soft and hard tissues surrounding implants, categorized into peri-implant mucositis (PM) and peri-implantitis (PI). PM refers to inflammation of the mucosa around the implant without associated supporting bone loss. In contrast, PI encompasses both mucosal inflammation and bone loss [1]. In 2015, the incidence of PM and PI was 19% to 65% and 1% to 47%, respectively [2]. Moreover, it was observed that the incidence of peri-implant disease increased with the prolonged duration of implant restoration [3]. Therefore, it is imperative to investigate new methods and optimal strategies for the prevention and treatment of peri-implant disease in clinical research. This is of paramount importance for effectively enhancing the rate of dental implant retention.

The principle of treating peri-implant disease involves removing plaque and dental calculus, controlling inflammation, and promoting periodontal tissue adhesion through non-surgical and surgical treatments. PM and mild PI are usually managed with non-surgical methods, while moderate and severe PI often require non-surgical treatments combined with regenerative surgery to control inflammation and repair bone loss. Generally, mechanical debridement (MD) is considered the primary treatment for peri-implant disease, primarily involving supramarginal debridement for the treatment of PM and submarginal debridement for the treatment of PI [8]. However, MD alone may not be effective in removing calculus and soft tartar at the junction of the implant and superstructure. Additionally, repeated friction of the implant with mechanical tools can lead to corrosion of the chemical oxide layer on the implant surface or the detachment of titanium particles, affecting implant biocompatibility and ultimately increasing inflammation [4, 5]. In the past decade, scholars have used lots of non-surgical methods combined with MD in the clinical treatment of peri-implant disease.

Among these therapies, various lasers and photodynamic treatments have gained widespread use as adjunctive therapies for periodontal and peri-implant diseases. Laser therapy, for instance, can eliminate bacteria by evaporating water through a thermo-mechanical effect, creating intense pressure and causing micro-explosions. Importantly, when used correctly, lasers do not alter the surface morphology of the implant [6, 7]. Photodynamic therapy (PDT) involves the use of a photosensitizer and a specific laser light wavelength to create reactive oxygen species (ROS) with the involvement of oxygen. These ROS interact with bacteria, leading to a super-oxidizing reaction that effectively eliminates pathogenic bacteria [8, 9]. Photobiomodulation therapy (PBMT), on the other hand, enhances microcirculation, cell proliferation, and neurotransmission [10]. When combined with MD, it has been found to reduce the expression of proteases (e.g., tissue fibrinogen activator) in gingival sulcus fluid [11]. This combination also significantly decreases peri-implant bleeding on probing (BoP) in the short term [12]. Air-powder polishing (APP) and probiotics therapy have demonstrated efficacy in addressing plaque biofilm. APP involves the use of glycine- or erythritol-based powders in compressed air to remove biofilm, potentially improving the effectiveness of non-surgical PI treatment [13]. Probiotics play a modulating role in oral biofilm components and notably reduce pro-inflammatory cytokine responses, resulting in improved clinical parameters for most patients with PI [14]. Additionally, systemic antibiotics (SA), such as metronidazole and tetracycline, and local antimicrobials (LA), can effectively target Gram-negative bacteria in peri-implant diseases. These treatments have direct anti-inflammatory effects on local peri-implant diseases.

In modern medicine, MD alone often falls short of the efficiency and minimally invasive criteria in treating peri-implant diseases. There’s a growing trend to combine non-surgical treatments with MD for better results. However, the efficacy of different non-surgical combinations varies, necessitating the exploration of optimal treatment combinations and understanding differences in effectiveness to guide clinical practice. Previous meta-analyses typically compared only two treatment groups, neglecting the comprehensive effects of various methods. Furthermore, they often overlooked statistical analysis of plaque index (PLI), clinical attachment level (CAL), and marginal bone loss (MBL), which are essential for assessing antimicrobial efficacy and visualizing tissue healing. These factors reflect treatment effectiveness from multiple angles, making a systematic analysis necessary.

To comprehensively and systematically evaluate and compare clinical outcomes and differences between various non-surgical approaches (Laser, PBMT, PDT, SA, probiotics, LA, APP) combined with MD for peri-implant disease, our group conducted this network meta-analysis (NMA). This NMA aims to provide theoretical and statistical guidance for selecting the optimal clinical treatment plan.

## Materials and methods

The NMA were conducted according to the Cochrane Handbook for Intervention Reviews and Preferred Reporting Items for Meta-Analyses (PRISMA) statement [15]. The study protocol is published in the Prospective Register of Systematic Reviews (PROSPERO); ref CRD 42023447616.

### Search strategy

As of July 25, 2023, two independent reviewers (Y.B. and S.Q.) conducted a literature search. Four electronic databases, including PubMed, Embase, Web of Science, and The Cochrane Library, were screened to identify relevant manuscripts. The search utilized a combination of MeSH terms, keywords, and free text terms, such as "peri-implant disease", "peri-implantitis", "periimplantitis", "peri-implant mucositis", “periimplant mucositis", "non-surgical", "nonsurgical", "treatment", “randomized controlled trial”, “randomized” and “placebo”. Additionally, references in relevant papers and reviews were manually cross-checked to identify additional articles. For detail information on the search strategy see S1 File.

### Inclusion and exclusion criteria

Inclusion criteria were defined as follows: (i) each patient (aged at least 18 years) had at least one dental implant suffering from peri-implantitis or peri-implant mucositis in accordance with the 2018 Classification of Periodontal and Peri-implant Diseases and Conditions [16–18]; (ii) the intervention group received any type of non-surgical treatment in combination with MD treatment; (iii) the control group received MD treatment alone; (iv) primary outcome: pocket probing depth (PPD), secondary outcome: bleeding on probing (BoP), plaque index (PLI), clinical attachment level (CAL), and marginal bone loss (MBL); (v) Only randomized controlled trial (RCT) treating at least 5 patients per group was considered; (vi) Minimum follow-up was 3 months. Exclusion criteria were defined as: (i) animal and in vitro studies; (ii) surgical approaches; (iii) subjects with systemic diseases; (iv) all subjects being smokers; (v) interventions with unclear outcome measures, missing data, or unusable literature; (vi) non-RCT.

### Study selection and data collection

Two researchers (Y.B. and S.Q.) independently conducted the literature search and screening based on the established inclusion and exclusion criteria. In cases of disagreement, a third researcher (B.L.) facilitated discussions. The extracted information included the first author, publication date, country, study type, intervention, sample size, follow-up duration, outcome indicators, and main results.

### Quality assessment

For RCTs, the risk of bias was assessed using the Cochrane Collaboration Risk of Bias Tool [19]. This assessment covered several domains, including random sequence generation, allocation concealment, blinding of participants and personnel, blinding of outcome assessment, incomplete outcome data, selective reporting, and other biases. For each domain, we will grade methodological quality as ‘low risk,’ ‘unclear risk,’ or ‘high risk’ according to the Cochrane Handbook version 5.1.0. If none of the domains is rated as “high risk”, studies will be classified as “low overall risk” or three or fewer are rated as “unclear risk”; moderate if one is rated as ‘high risk’ or none is rated as “high risk” but four or more are rated as “unclear risk” and otherwise as “high overall risk”.

### Data analysis

We began with a two-by-two meta-analysis comparing various interventions against a control group to assess their effects on all outcomes. In traditional meta-analyses, we measured inter-study heterogeneity using the i^2^ statistic, categorizing it as mild, moderate, or high heterogeneity at 25%, 50%, and 75%, respectively [29]. When heterogeneity was substantial (i^2^ ≥ 50%), we employed a random-effects model. We conducted network meta-analyses using frequency models, combining both direct and indirect evidence from all available studies. These analyses were performed using STATA 17.0 software (STATA, College Station, TX, United States) within the Network package, allowing us to account for clinical and other study factors that may introduce heterogeneity and provide more conservative confidence intervals (CI) for merged point estimates. Comparative relationships between intervention groups were illustrated through network diagrams. Effect sizes were estimated using the standardized mean difference (SMD) and 95% confidence intervals (95% CI). Effect sizes were categorized based on Cohen’s criteria, with |0.8| or greater indicating a large effect, |0.5| to < |0.8| a medium effect, |0.2| to < |0.5| a small effect, and less than |0.2| considered trivial. To ensure that multiple treatment comparisons were sufficiently similar, we assessed network transmissibility by comparing clinical and methodological features. Consistency was evaluated through treatment interaction models (global approach), node-split tests (i.e., local approach), and consistency models to identify significant differences between direct and indirect comparisons for each treatment. We used the surfaces under a cumulative ranking (SUCRA) curve to summarize probability values. SUCRA values ranged from 0 (worst treatment) to 1 (best treatment). Additionally, we conducted sensitivity analyses, excluding trials of poor and fair quality, to evaluate the stability of the results. Publication bias and small study effects were assessed through funnel plots.

## Results

### Literature search

**Fig 1:**
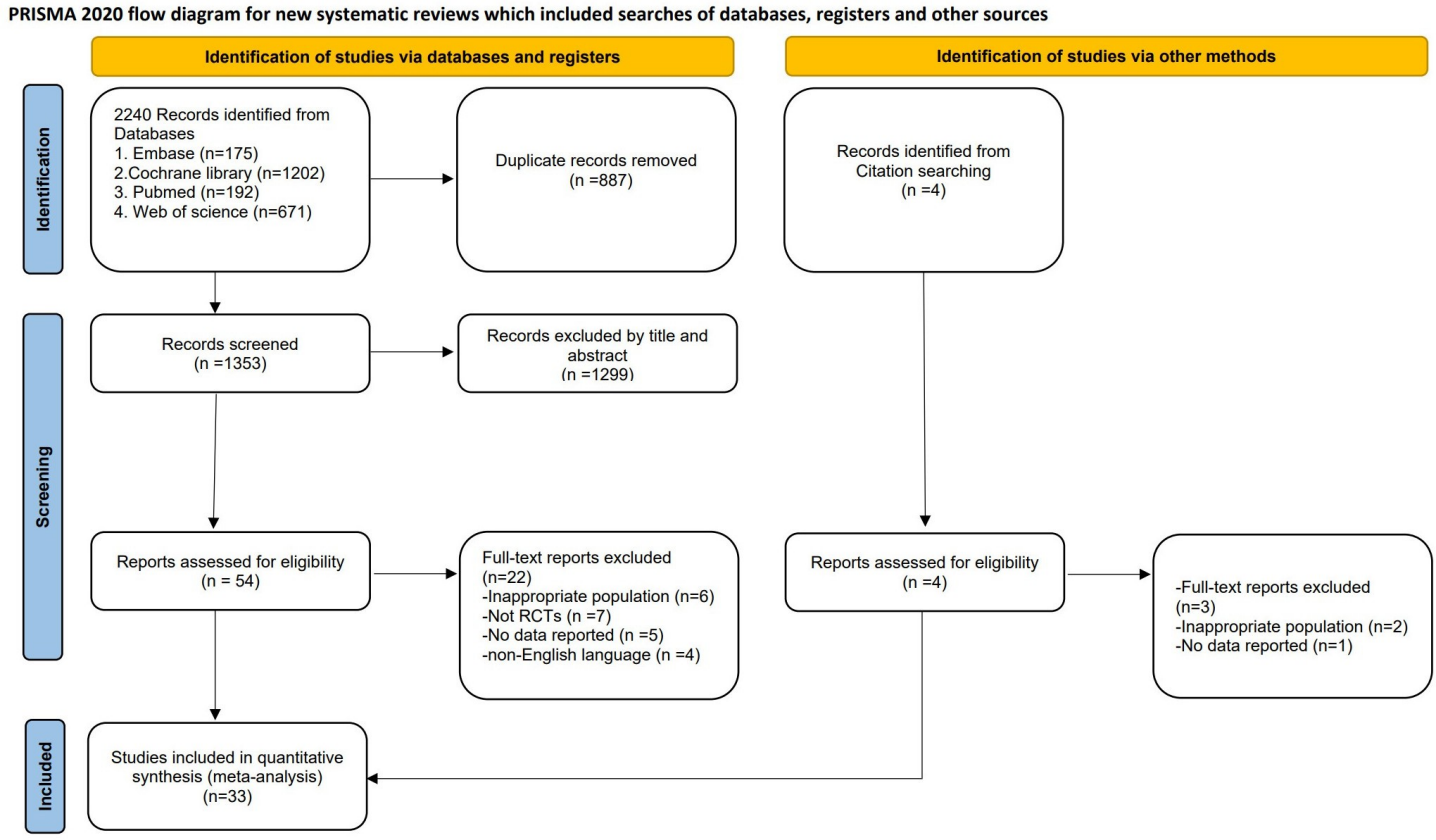
PRISMA flow diagram of the study selection criteria.

We conducted a literature search, and Fig 1 illustrated the flow chart of our retrieval process. Our search strategy initially identified 2,244 studies from electronic databases and manual checks of reference lists. Of these, 887 were duplicates and subsequently excluded. After screening titles and abstracts based on our inclusion and exclusion criteria, 1,299 records were excluded. We then conducted a detailed review of the remaining 58 articles. In the end, 33 articles met the selection criteria for both phases and were included in this study.

### Characteristics of included studies

**Fig 2:**
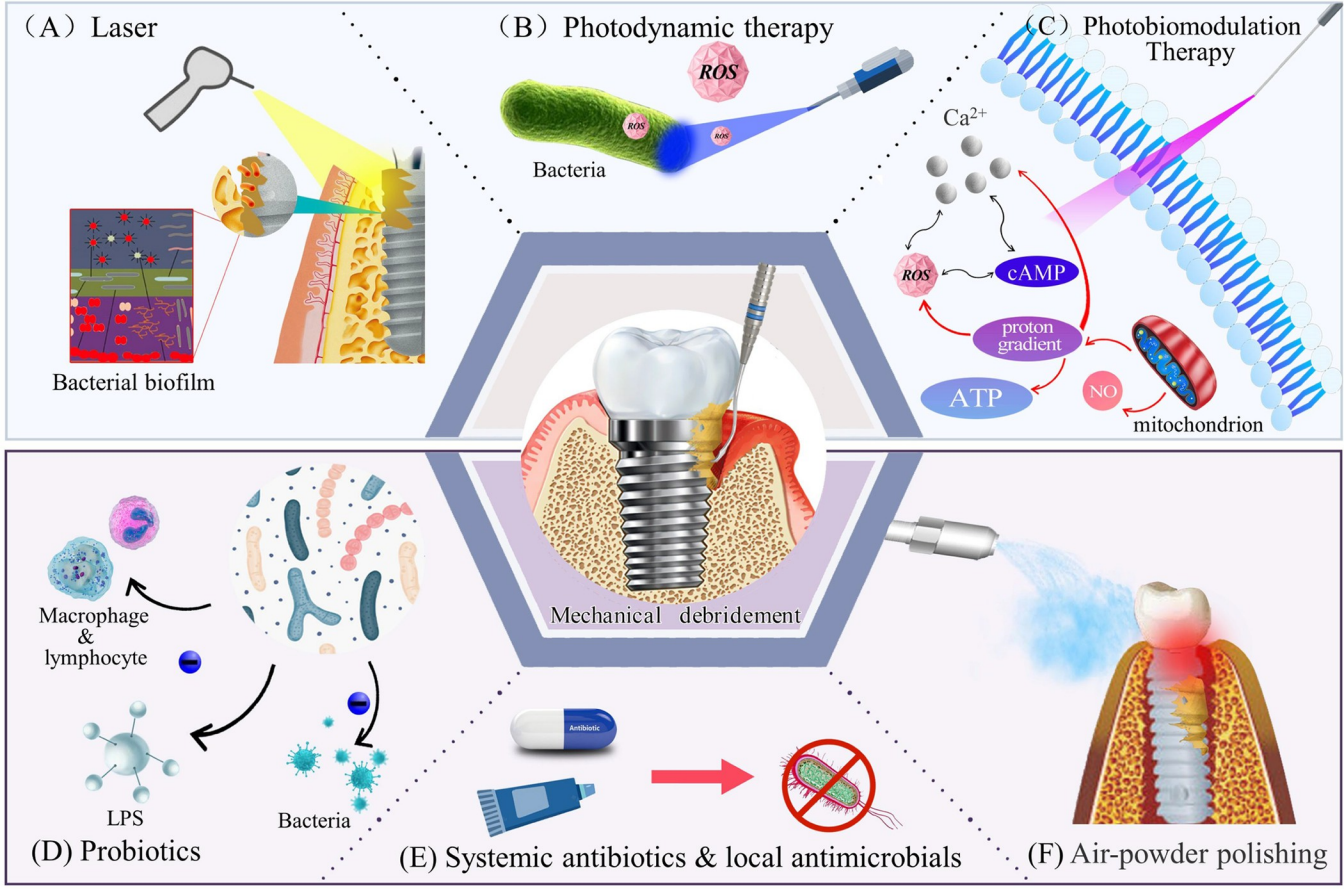
Overview of seven non-surgical methods combined with mechanical debridement in the treatment of peri-implant diseases.

The included studies can be categorized into seven treatment combinations, as shown in Fig 2. Tables 1 and 2 summarize the basic characteristics of these 33 human clinical trials published between 2004 and 2023. These studies encompassed seven therapeutic models: MD+ laser (8 items), MD+PBMT (2 items), MD+PDT (3 items), MD+SA (6 items), MD+ probiotics (4 items), MD+LA (10 items), and MD+APP (3 items). Of the included studies, 32 reported changes in PPD, 15 reported changes in BoP, 9 reported changes in PLI, 7 reported changed in CAL, and6 reported changes in MBL.

**Table 1:** Summary table of meta-analyses comparing clinical efficacy of different non-surgical therapy combined with mechanical debridement in treatment of peri-implantitis.

Study	Country	Design	Diagnosis	Groups	N° of patients /implants	Age (Years)	Follow-up	PPD (mm, SD)	BoP (%, SD)	PLI (%, SD)	CAL (mm, SD)	MBL (mm, SD)
Abduljabbar, 2017 [20]	Saudi Arabia	RCT	PI	T: MD+Laser (Nd:YAG) C: MD	T: 31 (35) C: 32 (39)	T:40.5±7.75 C:43.6 ±6.75	6 M	Baseline: T: 5.3±0.4, C:5.6 ± 0.5; 6months: T: 2.5 ± 0.25, C:4±0.43,	Baseline: T: 50.3±5.47, C:48.6 ± 4.03; 6months: T:10.5 ± 1.28, C:8.8 ± 0.85	Baseline C:52.5±2.8, T:57.3 ± 4.45; 6months: C:6.5 ± 0.83, T: 9.6 ± 1.48	-	Baseline C:1.8±0.43, T:2.1±0.3; 6months: C:1.7±0.35, T: 2.2±0.3
Al-Askar, 2022 [21]	Saudi Arabia	RCT	PI	T1: MD+PBMT T2: MD+PDT C: MD	T1: 17 (17) T2: 16 (16) C: 16 (16)	T1:63.2±2.8 T2: 65.2 ± 1.3 C: 62.8 ± 2.5	3 M	Baseline: T1: 4.5±0.3, T2:4.2 ± 0.1 C:4.4 ±0.2; 3 months: T1: 2.3±0.3, T2: 1.4±0.07, C: 2.7 ±0 .03	-	-	-	-
Almohareb, 2020 [22]	Saudi Arabia	RCT	PI	T1: MD+PDT T2: MD+SA (AMX+MTZ)	T1: 20 (43) T2: 20 (36)	T1: 51.7 ± 7.5 T2: 50.9 ± 6.3	12 M	Baseline: T1: 5.2 ± 2.0, T2: 5.4 ± 2.1; 12months: T1: 3.8 ± 0.9, T2: 4.1 ± 1.0	Baseline: T1: 45.3 ± 14.8, T2: 43.8 ± 13.9; 12months: T1: 18.6 ± 7.9, T2: 25.7 ± 8.1	-	-	-
Alpaslan,2021 [23]	Turkey	RCT	PI	T1: MD +Laser (Er,Cr:YSGG) T2:MD+Laser (Diode) C: MD	T1: 17 (17) T2: 16 (16) C: 17 (17)	T1:54.7±7.34 T2:46.5±11.34 C:50.36±6.85	6 M	Baseline: T1:4.48±1.14, T2: 4.14±0.80, C: 4.14 ± 0.64; 6months: T1: 3.33 ±0.93, T2: 3.28±1.00, C: 3.62±0.71	Baseline: T1: 100.00±0.00, T2: 88.09 ± 17.82, C: 72.02 ± 23.93; 6months: T1: 51.19 ± 19.84, T2: 61.90±29.37, C: 60.71±29.13	-	-	-
Arisan, 2015 [24]	Turkey	RCT	PI	T:MD+Laser (Diode) C: MD	T: 5 (24) C: 5 (24)	55.1 (43–76)	6 M	Baseline: T: 4.71 ±0.67, C:4.38 ±0.42; 6months: T: 4.54 ±0.74, C: 4.17 ± 0.41	-	-	-	Baseline: T: 2.13 ± 0.47, C: 2.35 ±0.56; 6months: T: 2.79 ± 0.48, C: 2.63±0.53,
Blanco, 2022 [25]	Spain	RCT	PI	T: MD+SA (MTZ) C: MD	T: 16 (34) C: 16 (28)	T:58.31 (51.46–65.16) C:60.74 (55.09–65.84)	12 M	Baseline: T:6.78±0.42, C: 6.32 ±0.31; 12months: T:4.34±0.29, C: 5.43 ±0.31	-	-	Baseline: T:7.24 ± 0.41, C: 6.59 ± 0.26; 12months: T:5.51 ± 0.30, C: 6.09 ±0.31	Baseline: T: 6.31 ± 0.38, C: 6 ± 0.41; 12months: T: 4.16 ± 0.39, C:5.05 ± 0.43
Büchter, 2004 [26]	Germany	RCT	PI	T:MD+LA(Doxycycline) C: MD	T: 14 (14) C: 14 (14)	T: 54 (25–61) C: 56 (38–78)	4 M	Baseline: T: 5.64 ± 0.32, C: 5.68 ±0.28; 4months: T: 4.49 ± 0.29, C: 5.4 ± 0.34	-	-	Baseline: T: 5.32 ± 0.33, C: 5.51 ± 0.27; 4months: T: 4.17 ± 0.30, C: 5.18 ± 0.33	-
Galofré, 2018 [27]	Spain	RCT	PI	T: MD+Probiotics C:MD	T: 11 (11) C: 11 (11)	T: 61.7 ± 7.0 C: 56.8 ± 9.3	3 M	Baseline: T: 5.07 ± 0.87, C: 4.90 ± 0.66; 3months: T: 4.53 ± 0.72, C: 4.70 ± 0.75	-	-	-	-
Kang, 2022 [28]	USA	RCT	PI	T: MD+Laser (Er,Cr: YSGG) C: MD	T: 13 (26) C: 10 (38)	T: 62.53 ±7.12 C:67.19 ± 9.26	9 M	Baseline: T:4.9±0.23, C:4.7±0.15; 9months: T:3.6±0.23, C:4.0±0.18	Baseline: T:81±6.25, C:71±8; 9months: T:42±11, C:51±5.5	Baseline: T:55±11.25, C:64±7.75; 9months: T:26±8, C:34±8.25	Baseline: T:5.7±0.28, C:5.2±0.15; 9months: T:4.5±0.3, C:4.3±0.15	Baseline: T:3.80±0.31, C:3.37±0.20; 9months: T:3.85±0.30, C:3.39 ± 0.22
Machtei, 2012 [29]	Israel	RCT	PI	T: MD+ LA (CHX) C: MD	T: 26 (36) C: 30 (37)	T: 57.42 ±10.5 C: 60.95 ± 7.9	6M	Changes from baseline to 6months: T: -2.13±0.22, C: -1.73±0.19;	Changes from baseline to 6months: T: -57.5±7.92, C: -45.5±8.8	-	-	-
Machtei, 2021 [30]	Israel	RCT	PI	T: MD+LA (CHX) C: MD	T: 146 (197) C: 144 (189)	T: 62.5 ± 11.2 C: 62.6 ± 11.6	6 M	Baseline: T:6.16±1.00, C:6.06±0.92; 6 months: T:4.40±1.25 C:4.52±1.27	-	-	Baseline: T:6.66±1.31, C:6.32±1.11; 6months: T:5.20±1.73, C:4.94 ± 1.49	-
Merli, 2020 [31]	Italy	RCT	PI	T1:MD+APP (Desiccant material) T2:MD+APP (Glycine) T3:MD+APP (Desiccant material+glycine) C: MD	T1: 15(15) T2: 13(13) T3: 14(14) C: 16 (16)	T1: 60.3±10.7 T2: 66.4±9.4 T3: 60.3±8.5 C: 64.5±8.3	6 M	Baseline: T1:5.0±1.2, T2: 5.1 ± 1.5, T3:4.9 ± 1.1, C:4.4 ± 1.1; 6months: T1: 4.5±1.2, T2:4.8±1.3, T3:4.0 ± 1.2, C:4.2 ± 1.3	-	-	Baseline: T1:5.4±1.2, T2.5.4 ± 1.6, T3:5.0 ± 0.9, C: 4.4 ± 1.0; 6months: T1:4.9±1.3, T2: 5.2 ± 1.5, T3:4.0 ± 1.0, C:4.3 ± 1.3	-
Park, 2021 [32]	Korea	RCT	PI	T1:MD+LA(MIN+MTZ) T2: MD + LA (MIN) C: MD	T1: 39 (39) T2: 40 (40) C: 39 (39)	T1: 60.7 (45–77) T2: 61.2 (40–76) C:61.2(41–77)	3 M	Changes from baseline to 3months: T1: -1.95±1.28, T2: -1.88±1.50, C: -1.28±1.15	Changes from baseline to 3months: T1: -8.5±5.3, T2: -8.3±5.6, C: -5.5±6.8	-	-	-
Polymeri, 2022 [33]	Netherlands	RCT	PI	T: MD+SA (AMX+MTZ) C: MD	T: 18 (18) C: 19 (19)	T:58.3±13.9 C:60.8±14.8	3M	Changes from baseline to 3months: T: -1.47± 1.95, C: -2.28±1.49	-	-		
Roccuzzo, 2022 [34]	Switzerland	RCT	PI	T: MD+Laser (Diode) C: MD	T: 12 (12) C: 13 (13)	T: 67.3 ± 12.2 C: 61.0 ± 13.2	6 M	Baseline: T:5.40± 0.81, C: 5.29±0.52; 6months: T: 4.13±0.82, C: 3.82 ± 0.88	Baseline: T:62.5 ± 30.3, C: 62.8 ± 21.7; 6months: T: 47.2 ± 33.2, C: 47.4 ± 27.9	Baseline: T:8.3 ±16.7, C: 3.8 ±10.0; 6months T: 9.7±13.2, C: 10.3±27.7	-	-
Roos-Jansaker, 2017 [35]	Sweden	RCT	PI	T: MD+LA (Chloramine) C: MD	T: 16 (16) C: 16 (16)	72.0±7.0	3 M	-	Changes from baseline to 3months: T: -9.9±6.3, C: -11±5.5	-	-	-
Selimovic, 2023 [36]	Norway	RCT	PI	T: MD +APP (Erythritol) C: MD	T: 23 (31) C: 20 (31)	T: 65.8±11.6 C: 64.5±13.6	12 M	Baseline: T: 4.5±0.10, C: 4.4±0.10; 12months: T: 4.2±0.11, C: 3.8±0.09	-	-	-	Baseline: T: 3.6±0.22, C: 3.1±0.20; 12months: T: 3.5±0.21, C: 3.4±0.27
Shibli, 2019 [37]	Brazil	RCT	PI	T: MD+SA (AMX+MTZ) C: MD	T: 20 (20) C: 20 (20)	58.5±11.1	12 M	Baseline: T: 7.0 ± 2.6, C: 5.5 ± 1.3; 12months: T: 3.9 ± 0.8, C: 3.8 ± 1.1	-	-	Baseline: T: 7.2 ± 2.6, C: 5.9 ± 1.3; 12months: T: 4.2 ± 1.0, C: 4.4 ± 1.4	-
Strauss, 2021 [38]	USA	RCT	PI	T: MD+Laser (Nd:YAG) C: MD	T: 10 (19) C: 10 (15)	-	12 M	Changes from baseline to 12months: T: -1.89±1.33, C: -1.36±2.01	-	-	-	Changes from baseline to 12months: T: -0.41 ± 0.92, C: -0.26 ± 0.64
Wang, 2019 [39]	China	RCT	PI	T: MD+PDT C: MD	T: 65 (65) C: 66 (66)	T: 44.1±9.8 C: 42.6±13.0	6 M	Baseline: T: 4.93± 1.07, C 5.07 ± 0.72; 6months: T: 3.06 ± 0.29, C: 4.62± 0.45	-	-	Baseline: T: 1.85 ± 0.86, C:1.32±0.43; 6months: T: 1.32 ± 0.43, C:1.32±0.43	-

PI, peri-implantitis; Nd: YAG, neodymium-doped yttrium aluminum garnet; MD, mechanical debridement; PPD, pocket probing depth; BoP: bleeding on probing; PLI, plaque index; CAL, clinical attachment level; MBL, marginal bone loss; PBMT, photobiomodulation therapy; PDT, photodynamic therapy; SA, systemic antibiotics; AMX, amoxicillin; MTZ, metronidazole; Er,Cr:YSGG, erbium.chromium:yttrium scandium gallium garnet; LA, local antimicrobials; CHX, chlorhexidine; APP, air-powder polishing; MIN, minocycline

**Table 2:** Summary table of meta-analyses comparing clinical efficacy of different non-surgical therapy combined with mechanical debridement in treatment of peri-implant mucositis.

Study	Country	Design	Diagnosis	Groups	N° of patients /implants	Age (Years)	Follow-up	PPD (mm, SD)	BoP (%, SD)	PLI (%, SD)
Alhumaidan, 2022 [40]	Saudi Arabia	RCT	PM	T: MD+PBMT C: MD	T: 17(17) C: 17 (17)	T: 46.1± 6.5: C: 50.2± 2.7	4 M	Baseline: T:5.5 ± 0.6, C:5.1 ± 0.3; 4 months T:1.4 ± 0.1 C:1.5 ± 0.06	-	-
Alqahtani, 2021 [41]	Saudi Arabia	RCT	PM	T1: MD+Probiotics T2:MD+SA (AMX) C: MD	T1: 14 (14) T2: 14 (14) C: 14 (14)	T1: 45.2±5.1 T2: 45.3±4.9 C: 46.1±3.7	6 M	Baseline: T1: 5.2±0.5, T2:5±0.6 C:5.2±0.5; 6 months: T1: 1.21±0.16, T2: 2.61±0.39, C: 3.79±0.59	Baseline: T1: 48.6±6.6, T2: 46.2±5.4, C:48.7±3.6; 6 months: T1: 33.24±8.63, T2: 31.96±8.31, C: 44.43±10.87	Baseline: T1: 42.2±9.2, T2:40.6 ± 7.3 C:40.5±8.1; 6 months: T1: 22.05 ±4.3, T2: 24.93 ± 4.48, C: 36.44 ± 6.71
Bollain, 2021 [42]	Spain	RCT	PM	T: MD+LA (CHX+CPC) C: MD	T: 24 (24) C: 22 (22)	NR	12 M	Baseline: T: 2.45 ±0.56, C: 2.63 ±0.26; 12months: T:2.41±0.40, C: 2.54±0.32	Baseline: T: 29.95 ± 12.30, C: 27.65±9.60; 12months: T: 5.50 ± 5.75, C: 7.98 ± 7.55	-
Galofré, 2018 [27]	Spain	RCT	PM	T: MD+Probiotics C:MD	T: 11 (11) C: 11 (11)	T: 61.5 ± 10.4 C: 60.0 ± 9.5	3 M	Baseline: T: 3.84 ± 0.55, C: 3.82 ± 0.64; 3months: T: 3.35 ± 0.76, C: 3.66 ± 0.62	-	-
González, 2021 [43]	Spain	RCT	PM	T: MD+LA (Mixed gel) C: MD	T: 23 (23) C: 23 (23)	T:61.83±13.32 C:60.39±11.98	3 M	Baseline: T:3.86 ±0.79; C: 3.7± 0.7; 3months: T: 0.85± 0.63 C:0.46± 0.44	Baseline: T: 59.42±24.66; C: 61.23±20.81; 3months: T: 31.64 ±26.84 C: 48.86 ±21.56	Baseline: T: 35.46 ± 31.23; C: 25.94 ± 27.20; 3months: T: 7.6 ±11.49 C: 12.5 ±14.72
Hallström, 2016 [44]	Sweden	RCT	PM	T: MD+Probiotics C: MD	T: 24 (24) C: 25 (25)	T: 53.7±19.6 C: 63.3±17.2	6 M	Baseline: T:4.3 ± 1.1, C:4.0 ± 1.4; 6months: T:3.7±1.3, C:3.5±1.5	-	-
Hallström, 2012 [45]	Sweden	RCT	PM	T: MD+SA (AZM) C: MD	T: 22 (22) C: 21 (21)	T: 54.6± 18.2 C: 54.6± 19.8	6 M	Baseline: T:4.4±1.0, C:4.6±0.9; 6months T:3.5±1.1, C:4.1±1.2	Baseline: T:28.2±20.6, C:24.2 ±16.7;6months: T:10.1 ±6.9, C:18.4±17.4	Baseline: T:33.7±35.0, C:22.0±29.2; 6months: T:6.8±13.8, C:17.9±28.7
Heitz, 2011 [46]	Australia	RCT	PM	T: MD+LA (CHX) C: MD	T: 14 (14) C: 15 (15)	T:57 C:53	3 M	Baseline: T: 3.68± 0,93, C: 3.6±0.85; 3months: T: 3.13 ± 0.93, C:2.98± 0.85	-	-
iorio-siciliano, 2019 [47]	Italy	RCT	PM	T: MD+LA (Hypochlorite) C: MD	T: 22 (33) C: 23 (34)	T:46.50±15.35 C:45.96 ± 9.84	6 M	Baseline: T:3.93±1.09, C:3.68±0.85; 6months: T:3.04 ± 0.46, C:3.07± 0.58	-	-
Ji, 2014 [48]	China	RCT	PM	T:MD+APP(Glycine) C: MD	T: 12 (17) C: 12 (16)	T:46.2 C:41.3	3 M	Baseline: T: 4.6±0.50, C:4.5±0.55; 3months: T:3.7±0.95, C:3.6±1.0	-	-
Mariani, 2020 [49]	Italy	RCT	PM	T: MD+Laser (Diode) C:MD	T: 38 (38) C: 35 (35)	T: 59.2 ± 9.3 C: 62.1 ± 6.8	12 M	Baseline: T:3.6 ± 0.7, C:3.8 ± 0.6; 12months: T: 3.1±0.7, C:3.3±0.6	Baseline: T:63.6±24.2, C:59.5±25.0; 12months: T:25.8±24.1, C:27.6±25.5	Baseline: T:49.6±20.7, C:44.8 ± 28.5; 12months, T:15.8 ± 14.9, C:18.6 ± 16.1
Menezes, 2016 [50]	Brazil	RCT	PM	T: MD+LA (CHX) C: MD	T: 22 (61) C: 15 (58)	57.4	6 M	Baseline: T:2.85 ± 0.60, C:2.72 ± 0.68; 6months: T:2.49 ± 0.60, C:2.49±0.67	Baseline: T:75.82±33.98, C:67.54±34.38; 6months: T:45.76±34.85, C:41.08±41.00	Baseline: T:38.52±34.02, C:52.15 ± 32.20; 6months, T:13.11±21.21, C: 12.06 ± 21.58
Sanchez-Martos, 2020 [51]	Spain	RCT	PM	T: MD+laser (Diode) C: MD	T: 34 (34) C: 34 (34)	T:59.16±12.09 C: 54.7±10.46	3 M	Baseline: T:1.277 ± 0.347, C:1.303 ± 0.409; 3months: T: 1.068±0.103, C: 1.166±0.263	-	-
Signorino, 2021 [52]	Italy	RCT	PM	T: MD+ Probiotics C: MD	T: 40 (40) C: 40 (40)	Women: 65±12 Men: 60±15	3 M	Baseline: T: 2.22 ± 0.86, C: 2.30 ± 0.77; 3months: T: 2.19 ± 0.76, C: 2.25 ± 0.63	Baseline: T: 40.42 ± 19.69, C: 34.19 ± 22.83; 3months: T: 33.30±16.43, C: 35.18±20.61	Baseline: T: 56.33 ± 22,96, C: 39.57±22.94; 3months: T: 42.04±19.48, C: 39.88± 19.77

PM, peri-implant mucositis; MD, mechanical debridement; PPD, pocket probing depth; BoP: bleeding on probing; PLI, plaque index; PBMT, photobiomodulation therapy; SA, systemic antibiotics; AMX, amoxicillin; LA, local antimicrobials; CHX, chlorhexidine; CPC, cetylpyridinium chloride; AZM, azithromycin; APP, air-powder polishing;

### Risk of bias

**Fig 3:**
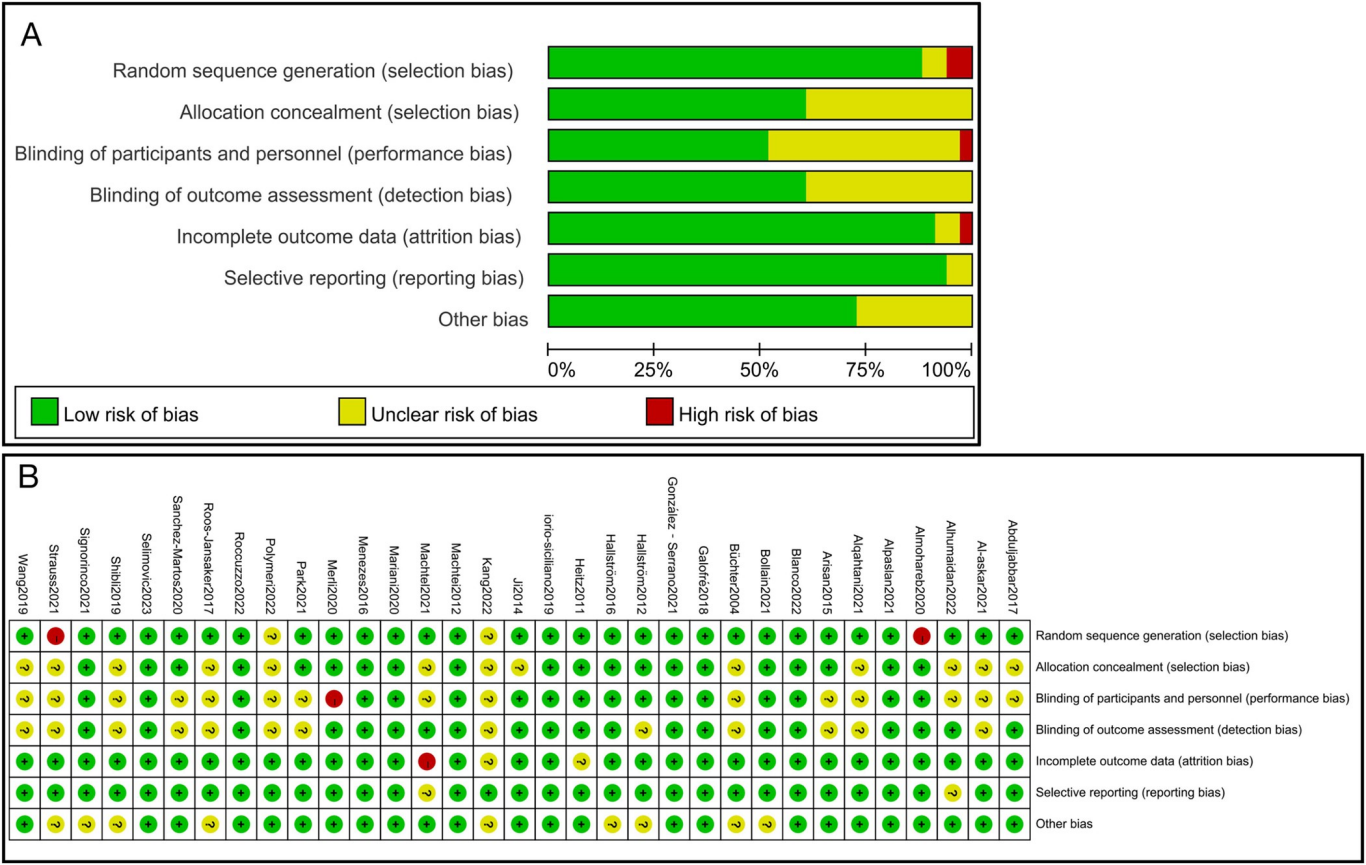
Risk of bias graph of the included studies.

The quality of the 33 included studies was assessed using the Risk of Bias Assessment Tool for RCTs in the Cochrane Handbook. Specifically, 29 studies detailed randomization methods, such as computerized tables of numbers and coin methods, while 20 studies described allocation concealment methods, such as envelopes and opaque containers. Overall, 10 studies were considered to have a low risk of bias in all domains, 4 were at high risk, and the remaining studies were at some risk of bias. Most of the included studies demonstrated high methodological quality. For specific quality and risk of bias evaluation results, please refer to Fig 3.

### Pocket probing depth (PPD) reduction

**Fig 4:**
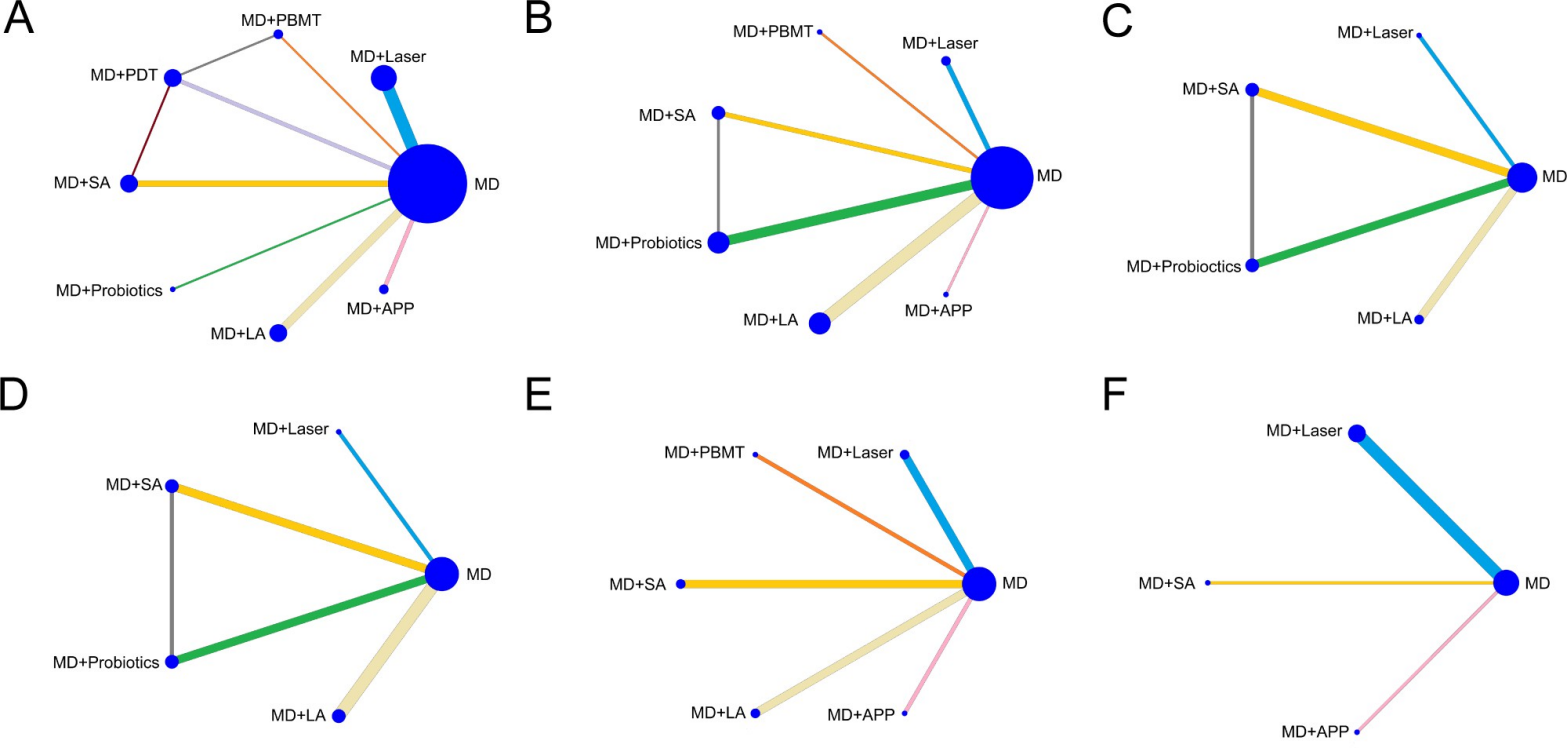
Network diagram for effect of each treatment. (A) PPD reduction for PI, (B) PPD reduction for PM, (C) BoP reduction for PM, (D) PLI reduction for PM, (E) CAL changes for PI, and (F) MBL changes for PI.

**Fig 5:**
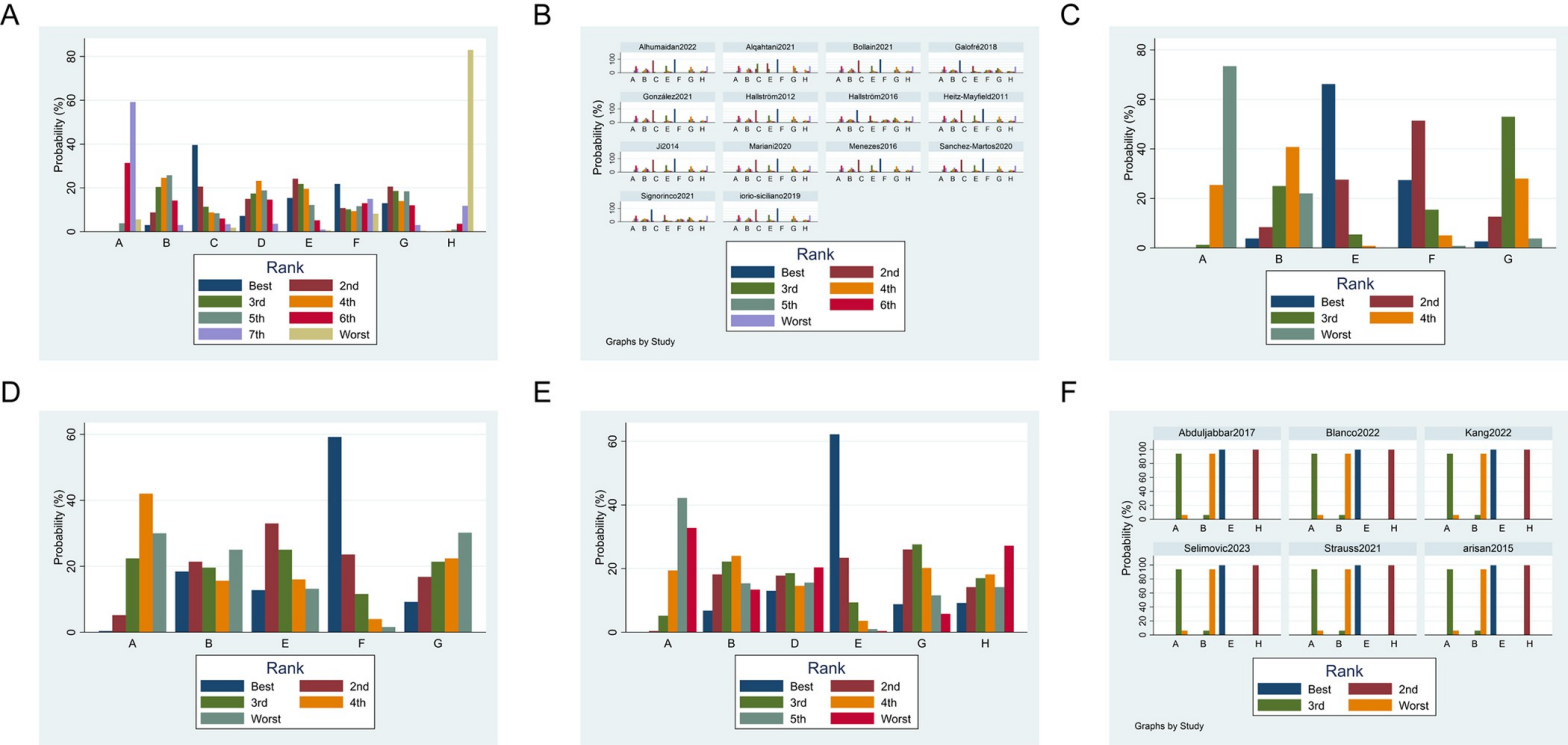
Ranking probability of each treatment. (A) PPD reduction for PI, (B) PPD reduction for PM, (C) BoP reduction for PM, (D) PLI reduction for PM, (E) CAL changes for PI, and (F) MBL changes for PI. Label: MD = A MD+ Laser = B MD+PBMT = C MD+PDT = D MD+SA = E MD+ probiotics = F MD+LA = G MD+APP = H.

In this study, 32 RCTs assessed the impact of seven different non-surgical treatments combined with MD on reducing PPD in peri-implant disease. For PI, overall inconsistency testing did not show significant inconsistency (p-value 0.71), indicating the use of a consistency model. The analysis formed two closed loops (Fig 4A), and a loop inconsistency test identified local inconsistency between direct and indirect comparisons involving MD/PDT+MD/SA+MD (inconsistency factor (IF) 0.59, 95% CI 0.00 to 3.52), with good agreement between MD/PBMT+MD/PDT+MD (IF 1.46, 95% CI 0.35 to 2.57), as shown in S1 Fig in S2 File. League table and forest plot revealed that compared with MD alone, SA+MD (SMD 1.45, 95% CI 0.12 to 2.78) and LA+MD (SMD 1.30, 95% CI 0.03 to 2.56) treatments for PPD reduction was statistically significant (p<0.05). Compared with APP+MD, laser+ MD (SMD 2.24, 95% CI 0.16 to 4.31), PBMT+MD (SMD 3.07, 95% CI 0.11 to 6.03), PDT+MD (SMD 2.42, 95% CI 0.06 to 4.77), SA+MD (SMD 2.68, 95% CI 0.44 to 4.92), LA+ MD (SMD 2.52, 95% 0.32 to 4.72) treatments for PPD reduction were statistically significant (p<0.05) (Table 3 and S2 Fig in S2 File). SUCRA analysis (Fig 5A and S1 Table in S2 File) ranked PBMT + MD as the top interventions (SUCRA = 75.3%).

**Table 3:** Standardized mean differences (SMDs) and 95% CI on PPD reduction of PI.

**MD+PBMT**							
0.39	**MD+SA**						
(-2.20, 2.98)							
0.55	0.16	**MD+LA**					
(-2.12, 3.22)	(-1.68, 2.00)						
0.65	0.26	0.11	**MD+PDT**				
(-1.70, 3.01)	(-1.44, 1.96)	(-1.87, 2.08)					
0.84	0.45	0.29	0.18	**MD+ laser**			
(-1.74, 3.41)	(-1.24, 2.14)	(-1.35, 1.93)	(-1.66, 2.02)				
0.91	0.52	0.36	0.26	0.07	**MD+ probiotics**		
(-2.61, 4.43)	(-2.42, 3.46)	(-2.55, 3.27)	(-2.77, 3.28)	(-2.74, 2.89)			
1.84	**1.45**	**1.30**	1.19	1.01	0.93	**MD**	
(-0.51, 4.20)	**(0.12, 2.78)**	**(0.03, 2.56)**	(-0.33, 2.71)	(-0.03, 2.05)	(-1.68, 3.55)		
**3.07**	**2.68**	**2.52**	**2.42**	**2.24**	2.16	1.23	**MD+APP**
**(0.11, 6.03)**	**(0.44, 4.92)**	**(0.32, 4.72)**	**(0.06, 4.77)**	**(0.16, 4.31)**	(-1.01, 5.34)	(-0.57, 3.03)	

*Lower left triangle refers to the SMD from the network meta-analysis. (eg, the SMD [95%CI] of PPD reduction between MD+PBMT and MD+APP is 3.07 [0.11, 6.03]). The data in bold indicates that the effect size is statistically significant (P<0.05).

For PM, global inconsistency testing indicated significant inconsistency between direct and indirect comparisons (p-value 0), necessitating the use of an inconsistency model. The analysis formed one closed loops (Fig 4B), and a loop inconsistency test identified local inconsistency between direct and indirect comparisons involving MD/SA+MD/probiotics+ MD (inconsistency factor (IF) 1.62, 95% CI 0.00 to 4.27), as shown in S3 Fig in S2 File. League table and forest plot revealed that compared with MD alone, PBMT+MD (SMD 1.11, 95% CI 0.38 to 1.84), SA+MD (SMD 1.03, 95% CI 0.02 to 2.03), LA+MD (SMD 3.59, 95% CI 2.43 to 4.76) and APP+MD (SMD 3.70, 95% CI 2.58 to 4.82) treatments for PPD reduction was statistically significant (p<0.05). Compared with laser+ MD, PBMT+MD (SMD 1.01, 95% CI 0.21 to 1.81), probiotics+ MD (SMD 3.60, 95% CI 2.43 to 3.77) treatments for PPD reduction was statistically significant (p<0.05). Compared with PBMT+MD, probiotics+ MD (SMD 2.59, 95% CI 1.25 to 3.93), LA+MD (SMD -0.90, 95% CI -1.66 to -0.13), APP+MD (SMD -1.11, 95% CI -2.11 to -0.11) treatments for PPD reduction were statistically significant (p<0.05). Compared with SA+MD, probiotics+ MD (SMD 3.33, 95% 2.06 to 4.60) treatments for PPD reduction were statistically significant (p<0.05). Compared with probiotics+ MD, LA+MD (SMD -3.49, 95% -4.63 to -2.34) and APP+MD (SMD -3.70, 95% -5.01 to -2.39) treatments for PPD reduction were statistically significant (p<0.05) (Table 4 and S4 Fig in S2 File). SUCRA analysis (Fig 5B and S2 Table in S2 File) ranked probiotics + MD as the top interventions (SUCRA = 100%).

**Table 4:** Standardized mean differences (SMDs) and 95% CI on PPD reduction of PM.

**MD+Probiotics**						
**2.59**	**MD+PBMT**					
**(1.25,3.93)**						
**3.33**	0.74	**MD+SA**				
**(2.06,4.60)**	(-0.20,1.69)					
**3.49**	**0.90**	0.15	**MD+LA**			
**(2.34,4.63)**	**(0.13,1.66)**	(-0.49,0.80)				
**3.60**	**1.01**	0.27	0.12	**MD+ laser**		
**(2.43,4.77)**	**(0.21,1.81)**	(-0.42,0.96)	(-0.28,0.52)			
**3.70**	**1.11**	0.37	0.22	0.10	**MD+APP**	
**(2.39,5.01)**	**(0.11,2.11)**	(-0.54,1.28)	(-0.50,0.93)	(-0.66,0.86)		
0.37	**1.11**	**1.03**	**3.59**	0.10	**3.70**	**MD**
(-0.23,0.97)	**(0.38,1.84)**	**(0.02,2.03)**	**(2.43, 4.76)**	(-0.23,0.43)	**(2.58,4.82)**	

*Lower left triangle refers to the SMD from the network meta-analysis. The data in bold indicates that the effect size is statistically significant (P<0.05).

### Bleeding on probing (BoP) reduction

In the analysis involving 7 RCTs for PM, the impact of four different non-surgical treatments combined with MD on BoP reduction was assessed and the network diagram is displayed in Fig 4C. The analysis formed one closed loop and identified local inconsistency between direct and indirect comparisons involving MD/SA+MD/probiotics+ MD (inconsistency factor (IF) 0.62, 95% CI 0.00 to 1.73), as is shown in S5 Fig in S2 File. League table and forest plot showed that SA+MD (SMD 0.77, 95% CI 0.27 to 1.28), probiotics+ MD (SMD 0.60, 95% CI 0.14 to 1.06) for BoP reduction were statistically significant compared to MD alone (p<0.05) (Table 5 and S6 Fig in S2 File). The SUCRA analysis (Fig 5C and S3 Table in S2 File) ranked SA+MD (SUCRA = 88.1%) as the top intervention, followed by probiotics+ MD (SUCRA = 75.7%).

**Table 5:** Standardized mean differences (SMDs) and 95% CI on BoP reduction of PM.

**MD+SA**				
0.17	**MD+ probiotics**			
(-0.42,0.77)				
0.46	0.28	**MD+LA**		
(-0.15,1.07)	(-0.26,0.83)			
0.57	0.40	0.11	**MD+ laser**	
(-0.18,1.32)	(-0.32,1.11)	(-0.54,0.77)		
**0.77**	**0.60**	0.32	0.2	**MD**
**(0.27,1.28)**	**(0.14,1.06)**	(-0.04,0.67)	(-0.34, 0.75)	

*Lower left triangle refers to the SMD from the network meta-analysis. The data in bold indicates that the effect size is statistically significant (P<0.05).

### Plaque index (PLI) reduction

A total of 6 RCTs comparing the effect of 4 non-surgical therapies in combination with MD on PLI reduction for PM were included. Overall inconsistency test showed a p-value of greater than 0.05 for the reduction in PLI after treatment, which could be analyzed by consistency model. As shown in Fig 4D, the included literature formed 1 closed loop and the loop inconsistency test identified local inconsistency between direct and indirect comparisons involving MD/SA+MD/probiotics+ MD (inconsistency factor (IF) 1.22, 95% CI 0.1 to 2.35) (S7 Fig in S2 File). League table and forest plot showed that no statistical significance was observed in the two-by-two comparisons (p>0.05) (Table 6 and S8 Fig in S2 File). The SUCRA analysis (Fig 5D and S4 Table in S2 File) showed that probiotics+ MD (SUCRA = 83.2%) ranked the highest among these therapeutic interventions.

**Table 6:** Standardized mean differences (SMDs) and 95% CI on PLI reduction of PM.

**MD+Probiotics**				
1.07	**MD+SA**			
(-1.72,3.87)				
1.30	0.22	**MD+ laser**		
(-2.81,5.40)	(-3.88,4.33)			
1.63	0.55	0.33	**MD+LA**	
(-1.72,4.98)	(-2.79,3.90)	(-3.81,4.48)		
1.97	0.90	0.68	0.34	**MD**
(-0.36,4.31)	(-1.43,3.23)	(-2.70,4.06)	(-2.06, 2.75)	

*Lower left triangle refers to the SMD from the network meta-analysis.

### Clinical attachment level (CAL) changes

In the analysis involving seven studies that compared five non-surgical methods combined with MD in terms of CAL changes for PI, the overall inconsistency test indicated a p-value of greater than 0.05 for CAL changes (p-value 0.725), and the network diagram is shown in Fig 4E. League table and forest plot revealed that SA+MD (SMD 2.2; 95% CI 0.38 to 4.02) for CAL changes were statistically significant compared to MD alone (p<0.05), with no significant differences observed in other comparisons like laser+ MD (SMD 0.71, 95% CI -1.07 to 2.50), PDT+MD (SMD 0.75, 95% CI -1.75 to 3.24), probiotics+ MD (SMD 1, 95% CI -0.81,2.82), LA+MD (SMD 0.46, 95% CI -2.08 to 3.00) (p>0.05) (Table 7 and S9 Fig in S2 File). According to SUCRA analyses (Fig 5E and S5 Table in S2 File), SA+MD (SUCRA = 87.4%) achieved the best results among the five treatments.

**Table 7:** Standardized mean differences (SMDs) and 95% CI on CAL reduction of PI.

**MD+SA**					
1.20	**MD+LA**				
(-1.37,3.77)					
1.45	0.26	**MD+PDT**			
(-1.64,4.55)	(-2.83,3.34)				
1.49	0.29	0.03	**MD+ laser**		
(-1.07,4.04)	(-2.25,2.83)	(-3.04,3.10)			
1.74	0.55	0.29	0.26	**MD+APP**	
(-1.38,4.87)	(-2.57,3.67)	(-3.27,3.85)	(-2.85,3.36)		
**2.20**	1.00	0.75	0.71	0.46	**MD**
**(0.38,4.02)**	(-0.81,2.82)	(-1.75,3.24)	(-1.07,2.50)	(-2.08,3.00)	

*Lower left triangle refers to the SMD from the network meta-analysis. The data in bold indicates that the effect size is statistically significant (P<0.05).

### Marginal bone loss (MBL) changes

We examined the effects of three non-surgical approaches combined with MD on changes in MBL changes for PI in 6RCTs. The network diagram is displayed in Fig 4F. Global inconsistency testing indicated significant inconsistency between direct and indirect comparisons (p-value 0), necessitating the use of an inconsistency model. The NMA results showed there was significant difference between MD+SA and MD+APP (SMD 1.86, 95% CI 0.56 to 3.17), MD+ SA and MD (SMD 3.92, 95% CI 2.9 to 4.93), MD+APP and MD (SMD 2.05, 95% CI 1.24 to 2.87), MD+SA and MD+ laser (SMD 4.24, 95% CI 3.15 to 5.33), MD+APP and MD +laser (SMD 2.38, 95% CI 1.47 to 3.28) (p<0.05) (Table 8 and S10 Fig in S2 File). SUCRA analyses (Fig 5F and S6 Table in S2 File) ranked SA+MD (SUCRA = 99.9%) as the most effective intervention for improving MBL.

**Table 8:** Standardized mean differences (SMDs) and 95% CI on MBL reduction of PI.

**MD+SA**			
**1.86**	**MD+APP**		
**(0.56,3.17)**			
**3.92**	**2.05**	**MD**	
**(2.90,4.93)**	**(1.24,2.87)**		
**4.24**	**2.38**	0.32	**MD+ laser**
**(3.15,5.33)**	**(1.47,3.28)**	(-0.06,0.71)	

*Lower left triangle refers to the SMD from the network meta-analysis. The data in bold indicates that the effect size is statistically significant (P<0.05)

### Publication bias

**Fig 6:**
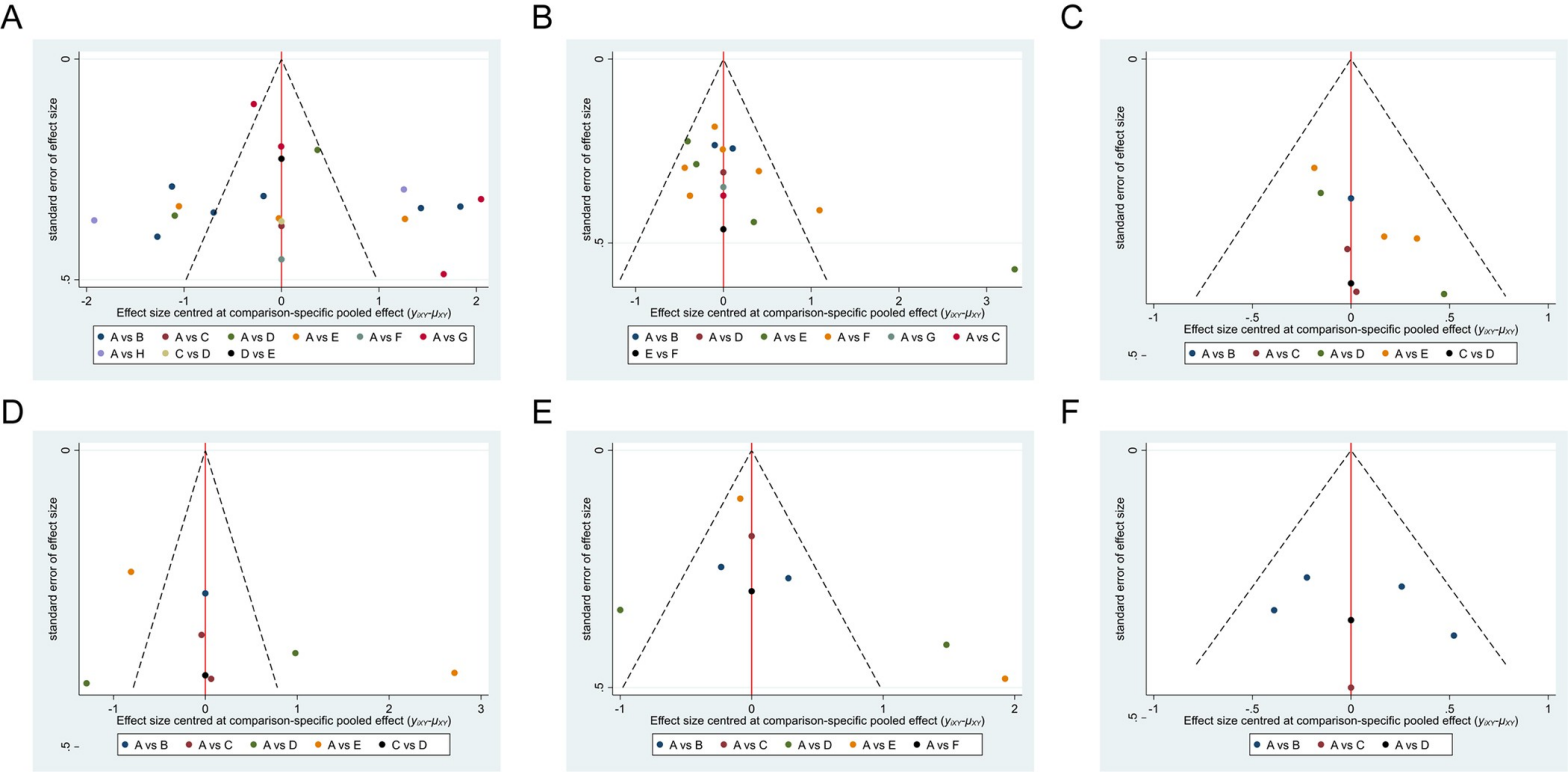
Funnel plot on publication bias. (A) PPD reduction for PI, (B) PPD reduction for PM, (C) BoP reduction for PM, (D) PLI reduction for PM, (E) CAL changes for PI, and (F) MBL changes for PI. Label: MD = A MD+ Laser = B MD+PBMT = C MD+PDT = D MD+SA = E MD+ probiotics = F MD+LA = G MD+APP = H.

Funnel plot was drawn for bias risk assessment. Visual asymmetry detection showed that the literature effect points formed an inverted funnel shape with basic symmetry, and most of the effect points were located at the top of the funnel plot, while only a few were outside the dashed line of the funnel plot, indicating that the quality of the included studies was good, publication bias was small, and the results were relatively reliable (Fig 6).

## Discussion

The clinical significance of this network meta-analysis is to rank the treatments by measuring the corresponding probabilities [49], estimating which treatment is most appropriate for each outcome. It allows us to make comparisons between several treatments that are not directly compared and to assess the effectiveness of each intervention, thus providing additional evidence for clinicians. Based on the results of our study, we have found that different combinations of interventions have their own merits in improving different outcomes, which helps us to select individualized treatment regimens based on clinical indicators for better clinical guidance. In addition, considering the limitations of certain treatments under certain conditions, such as the lack of medical equipment (lasers, photodynamic devices) or the limitations of the patient’s own condition (with antibiotic allergy or resistance), we can choose alternative therapies according to the ranking in order to treat the disease as much as possible.

### Peri-implantitis

Since there are few studies reporting BoP and PLI in the treatment of PI, involving only two or one treatment methods, it is of little significance to conduct a network meta-analysis for these two indexes. PPD is the distance from the mucosal margin to the bottom of the peri-implant pocket and is an important indicator of peri-implant soft tissue inflammation. According to the analysis of league table and SUCRA, PBMT+MD had the highest probability of ranking for reducing PPD in the analysis of PI. This was similar to the results obtained by Ren et al. in evaluating the effectiveness of PBMT treatment as an adjunct to nonsurgical periodontal therapy [53]. The conventional meta-analysis by Barbato et al. found SA+MD to be more effective on PPD compared to other interventions [54]. However, they did not include PBMT and other interventions in the analysis, so this may partly explain the inconsistency with our results.

CAL, representing the distance from the implant abutment junction to the bottom of the pocket, holds the key to assessing epithelial reattachment and periodontal tissue regeneration [55]. On the other hand, the MBL around the implant neck serves as a critical indicator for the long-term success of implant restorations and the maintenance of implant health. Barbato et al. conducted a meta-analysis on the clinical efficacy of non-surgical adjuvant treatment for PI. While their analysis focused on the indicators of PPD and BoP, our study ventured further by objectively analyzing CAL and MBL in cases of peri-implant disease [54]. Our findings revealed that after treatment with SA+MD, both CAL (SMD 2.2; 95% CI 0.38 to 4.2) and MBL (SMD 3.92, 95% CI 2.9 to 4.93) exhibited significant improvements compared with MD alone, ranking as the most effective interventions. This underscored the clinical value of SA, which consistently demonstrated notable anti-inflammatory effects and fostered an optimal metabolic environment for the healing of both hard and soft tissues. it’s important to note that although our study proved that systemic antibiotics provide effective help for peri-implant disease. However, according to 2023 Clinical S3-Guideline, it’s recommended against use in patients with peri-implant disease due to considerations of patient health and the impact of systemic antibiotic use to public health [56]. We recommended prioritizing the use of other adjunctive measures, especially for the treatment of PM and mild PI. Even if systemic antibiotics were chosen, the dosing interval should be controlled and narrow-spectrum antibiotics should be used whenever possible to minimize the risk of treatment.

### Peri -implant mucositis

When we conducted the analysis focusing on PM, the combination of probiotics with MD had the highest probability of ranking for reducing PPD and PLI. This implied that probiotics may have a more pronounced impact on addressing early inflammation. This was similar to the conclusions reached in the systematic review of MD combined with probiotics for periodontitis by Vives Soler et al [57]. According to the recently published (2023) Clinical S3-Guideline, probiotics taken under professional guidance in the treatment of peri-implant mucositis may be considered as adjunctive to MD [56]. This effectiveness may stem from probiotics’ inherent advantages in terms of nutrients and growth factors, which directly inhibited pathogen growth on the implant’s surface, preventing pathogen-mediated tissue damage and bolstering the host’s resistance to pathogens around the implant [58–61]. However, it’s important to note that the generalizability of probiotics in treatment of peri-implant diseases still require further observation and clinical evidence. Bleeding on probing (BoP) is also a measure of peri-implant soft tissue inflammation. Healthy peri-implant soft tissue does not usually bleed on light touch or probing [62]. Our study showed that SA+MD had the highest probability of ranking in reducing BoP for PM. However, according to 2023 Clinical S3-Guideline, it should be used with caution considering its side effects and drug dependence [56].

Our NMA has several limitations and shortcomings. Firstly, while we thoroughly investigated all RCTs with synthesizable data, there was still a lack of available trials with large sample sizes for direct comparison, which might have influenced the outcomes of our study. Secondly, we included studies with a minimum of 3 months follow-up (including 3, 6, and 12 months), which may have affected the outcome to some extent. This was because some treatments, such as Nd:YAG laser, can have a significant difference in the short term (3 months), while in the long term (6 months) the difference was not significant [20]. In addition, longer follow-up was needed for MBL because bone tissue took longer to heal compared to soft tissue. Moreover, due to the limited original data, we included a small number of studies related to CAL and MBL indicators and we were unable to assess additional clinical indicators, such as implant success rate, pain scores, and swelling grades. Finally, the included RCTs exhibited variations in study design, patient populations, diagnostic criteria, treatment methods, follow-up durations, and outcome measurements, making it challenging to draw definitive conclusions. As a result, it’s important to interpret the results of this NMA with caution.

## Conclusions

This is the first NMA outlining the efficacy of different non-surgical approaches combined with MD in peri-implant disease. The results have showed that different combination treatments have their own advantages in improving various clinical indicators of peri-implant disease. This study may be helpful in selecting individualized treatment regimens for different clinical indicators. However, despite the ranking established by our study for the treatment of peri-implant disease, decisions should still be made with reference to the latest treatment guidelines. There is still a need for more high-quality studies to provide conclusive evidence, especially regarding direct comparisons between multiple treatment options.

## Supporting information

S1 File Database and search strategy. (DOCX)

S2 File Further analysis. (DOCX)

S3 File PRISMA checklist. (DOCX)

## Abbreviations

MD: Mechanical debridement
PPD: Pocket probing depth
BoP: Bleeding on probing
PLI: Plaque index
CAL: Clinical attachment level
MBL: Marginal bone loss
SMD: Standardized mean difference
CI: Confidence interval
PM: Peri-implant mucositis
PI: Peri-implantitis
PDT: Photodynamic therapy
ROS: Reactive oxygen species
PBMT: Photobiomodulation therapy
APP: Air-powder polishing
SA: Systemic antibiotics
LA: Local antimicrobials
NMA: Network Meta-Analysis
RCT: Randomized controlled trial
SUCRA: Surfaces under a cumulative ranking
IF: Inconsistency factor
