# Supplementary material for: Comparison of the efficacy of seven non-surgical methods combined with mechanical debridement in peri-implantitis and peri-implant mucositis: A network meta-analysis
Source: PLoS One. 2024 Aug 14;19(8):e0305342. doi: 10.1371/journal.pone.0305342 (PMC11324115; doi:10.1371/journal.pone.0305342)
Supplement: S1 File — (DOCX) [file pone.0305342.s001.docx]

**Database and search strategy**

**PubMed on July 23, 2023**

| Search number | Query | Sort By | Filters | Search Details | Results |
| --- | --- | --- | --- | --- | --- |
| 6 | ((((((((((non-surgical therapy[Title/Abstract]) OR (nonsurgical therapy[Title/Abstract])) OR (nonsurgical treatment[Title/Abstract])) OR (Non-surgical treatment[Title/Abstract])) OR (treatment[Title/Abstract])) OR (therapy[Title/Abstract])) OR (non-surgical[Title/Abstract])) OR (nonsurgical[Title/Abstract])) NOT (surgical[Title/Abstract])) AND (("Peri-Implantitis"[Mesh]) OR (((((peri-implantitis[Title/Abstract]) OR (Periimplantitis[Title/Abstract])) OR (peri-implant mucositis[Title/Abstract])) OR (periimplant mucositis[Title/Abstract])) OR (peri-implant disease[Title/Abstract])))) AND (randomized controlled trial[Publication Type] OR randomized[Title/Abstract] OR placebo[Title/Abstract] OR random[Title/Abstract] OR randomised[Title/Abstract]) | | | (("non surgical therapy"[Title/Abstract] OR "nonsurgical therapy"[Title/Abstract] OR "nonsurgical treatment"[Title/Abstract] OR "non surgical treatment"[Title/Abstract] OR "treatment"[Title/Abstract] OR "therapy"[Title/Abstract] OR "non surgical"[Title/Abstract] OR "nonsurgical"[Title/Abstract]) NOT "surgical"[Title/Abstract]) AND ("peri implantitis"[MeSH Terms] OR ("peri implantitis"[Title/Abstract] OR "Periimplantitis"[Title/Abstract] OR "peri implant mucositis"[Title/Abstract] OR "periimplant mucositis"[Title/Abstract] OR "peri implant disease"[Title/Abstract])) AND ("randomized controlled trial"[Publication Type] OR "randomized"[Title/Abstract] OR "placebo"[Title/Abstract] OR "random"[Title/Abstract] OR "randomised"[Title/Abstract]) | 192 |
| 5 | randomized controlled trial[Publication Type] OR randomized[Title/Abstract] OR placebo[Title/Abstract] OR random[Title/Abstract] OR randomised[Title/Abstract] | | | "randomized controlled trial"[Publication Type] OR "randomized"[Title/Abstract] OR "placebo"[Title/Abstract] OR "random"[Title/Abstract] OR "randomised"[Title/Abstract] | 1,382,828 |
| 4 | ("Peri-Implantitis"[Mesh]) OR (((((peri-implantitis[Title/Abstract]) OR (Periimplantitis[Title/Abstract])) OR (peri-implant mucositis[Title/Abstract])) OR (periimplant mucositis[Title/Abstract])) OR (peri-implant disease[Title/Abstract])) | | | "peri implantitis"[MeSH Terms] OR "peri implantitis"[Title/Abstract] OR "Periimplantitis"[Title/Abstract] OR "peri implant mucositis"[Title/Abstract] OR "periimplant mucositis"[Title/Abstract] OR "peri implant disease"[Title/Abstract] | 4,319 |
| 3 | ((((peri-implantitis[Title/Abstract]) OR (Periimplantitis[Title/Abstract])) OR (peri-implant mucositis[Title/Abstract])) OR (periimplant mucositis[Title/Abstract])) OR (peri-implant disease[Title/Abstract]) | | | "peri-implantitis"[Title/Abstract] OR "Periimplantitis"[Title/Abstract] OR "peri implant mucositis"[Title/Abstract] OR "periimplant mucositis"[Title/Abstract] OR "peri implant disease"[Title/Abstract] | 4,073 |
| 2 | "Peri-Implantitis"[Mesh] | Most Recent | | "Peri-Implantitis"[MeSH Terms] | 2,077 |
| 1 | ((((((((non-surgical therapy[Title/Abstract]) OR (nonsurgical therapy[Title/Abstract])) OR (nonsurgical treatment[Title/Abstract])) OR (Non-surgical treatment[Title/Abstract])) OR (treatment[Title/Abstract])) OR (therapy[Title/Abstract])) OR (non-surgical[Title/Abstract])) OR (nonsurgical[Title/Abstract])) NOT (surgical[Title/Abstract]) | | | ("non surgical therapy"[Title/Abstract] OR "nonsurgical therapy"[Title/Abstract] OR "nonsurgical treatment"[Title/Abstract] OR "non surgical treatment"[Title/Abstract] OR "treatment"[Title/Abstract] OR "therapy"[Title/Abstract] OR "non surgical"[Title/Abstract] OR "nonsurgical"[Title/Abstract]) NOT "surgical"[Title/Abstract] | 5,913,294 |

**Embase on July 23, 2023**

| No. | Query | Results | Date |
| --- | --- | --- | --- |
| #6 | #3 AND #4 AND #5 | 175 | 24-Jul-23 |
| #5 | 'randomized controlled trial':ab,ti OR randomized:ab,ti OR placebo:ab,ti OR random:ab,ti OR randomised:ab,ti | 1649820 | 24-Jul-23 |
| #4 | ('non surgical':ab,ti OR nonsurgical:ab,ti OR treatment:ab,ti OR therapy:ab,ti) NOT surgical:ab,ti | 8182097 | 24-Jul-23 |
| #3 | #1 OR #2 | 4532 | 24-Jul-23 |
| #2 | periimplantitis:ab,ti OR 'peri implantitis':ab,ti OR 'peri-implant mucositis':ab,ti OR 'periimplant mucositis':ab,ti OR 'peri-implant disease':ab,ti | 3657 | 24-Jul-23 |
| #1 | 'periimplantitis'/exp OR 'periimplantitis' | 4331 | 24-Jul-23 |

**The Cochrane Library on July 23, 2023**

ID Search Hits

#1 MeSH descriptor: [Peri-Implantitis] explode all trees 303

#2 (peri-implantitis):ti,ab,kw OR (periimplantitis):ti,ab,kw OR (peri-implant mucositis):ti,ab,kw OR (periimplant mucositis):ti,ab,kw OR (peri-implant disease):ti,ab,kw (Word variations have been searched) 1956

#3 #1 OR #2 1956

#4 (non-surgical):ti,ab,kw OR (nonsurgical):ti,ab,kw OR (treatment):ti,ab,kw OR (therapy):ti,ab,kw NOT (surgical):ti,ab,kw (Word variations have been searched) 1178019

#5 #3 AND #4 1202

**Web of Science on July 25, 2023**

1: peri-implantitis (Topic) OR Periimplantitis (Topic) OR peri-implant mucositis (Topic) OR periimplant mucositis (Topic) OR peri-implant disease (Topic) and Preprint Citation Index (Exclude – Database) Date Run: Tue Jul 25 2023 00:17:04 GMT+0800 (GMT+08:00) Results: 8362

2: non-surgica (Topic) OR nonsurgical (Topic) OR treatment (Topic) OR therapy (Topic) NOT surgical (Topic) and Preprint Citation Index (Exclude – Database) Date Run: Tue Jul 25 2023 00:18:17 GMT+0800 (GMT+08:00) Results: 22012871

3: randomized controlled trial (Topic) OR randomized (Topic) OR placebo (Topic) OR random (Topic) OR randomised (Topic) and Preprint Citation Index (Exclude – Database) Date Run: Tue Jul 25 2023 00:19:41 GMT+0800 (GMT+08:00) Results: 3256854

4: #1 AND #2 AND #3 and Preprint Citation Index (Exclude – Database) Date Run: Tue Jul 25 2023 00:19:59 GMT+0800 (GMT+08:00) Results: 671
