# Supplementary material for: Comparison of the efficacy of seven non-surgical methods combined with mechanical debridement in peri-implantitis and peri-implant mucositis: A network meta-analysis
Source: PLoS One. 2024 Aug 14;19(8):e0305342. doi: 10.1371/journal.pone.0305342 (PMC11324115; doi:10.1371/journal.pone.0305342)
Supplement: S2 File — (DOCX) [file pone.0305342.s002.docx]

***Further analysis***

**S1 Fig: Inconsistency test (PPD reduction on peri-implantitis)**


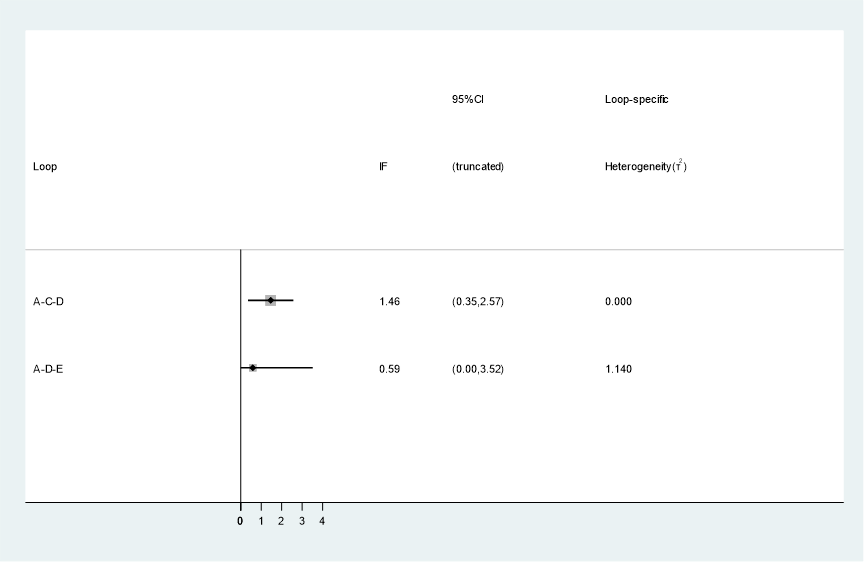


Label: MD=A MD+PBMT=C MD+PDT=D MD+SA=E

**S2 Fig: Forest plot (PPD reduction on peri-implantitis)**


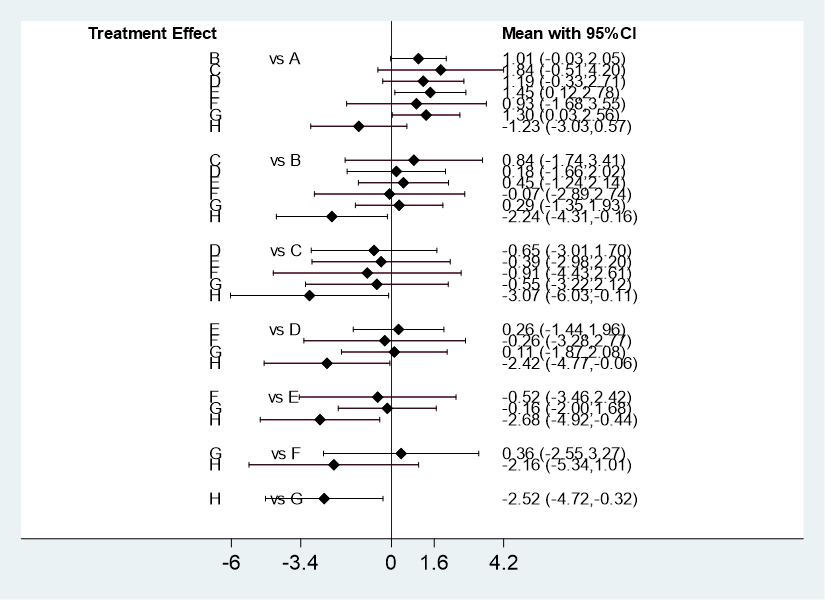


Label: MD=A MD+Laser=B MD+PBMT=C MD+PDT=D MD+SA=E MD+Probiotics=F MD+LA=G MD+APP=H

**S1 Table: SUCRA analysis: MeanRank table (PPD reduction on peri-implantitis)**

| treatment | SUCRA |
| --- | --- |
| A | 19.0 |
| B | 54.6 |
| C | 75.3 |
| D | 61.8 |
| E | 69.3 |
| F | 51.2 |
| G | 64.8 |
| H | 4.1 |

Label: MD=A MD+Laser=B MD+PBMT=C MD+PDT=D MD+SA=E MD+Probiotics=F MD+LA=G MD+APP=H

**S3 Fig: Inconsistency test (PPD reduction on peri-implant mucositis)**


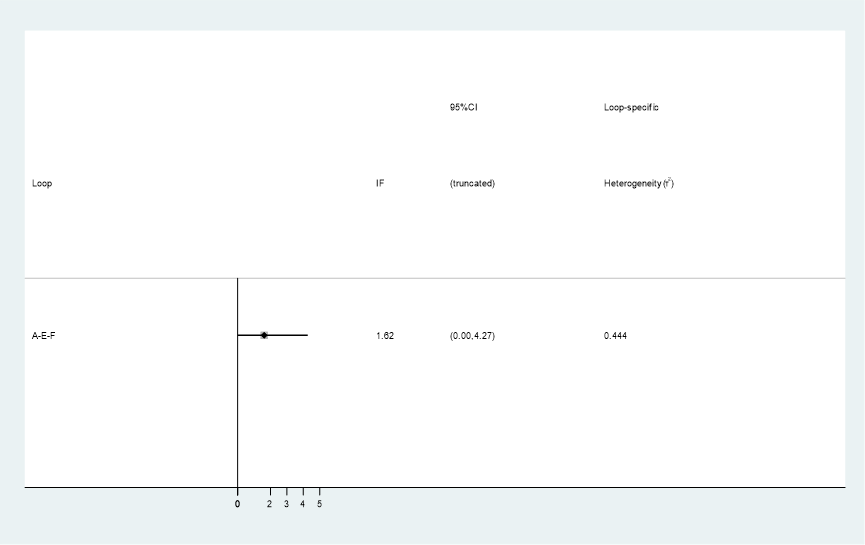


Label: MD=A MD+SA=E MD+Probiotics=F

**S4 Fig: Forest plot (PPD reduction on peri-implant mucositis)**

Label: MD=A MD+Laser=B MD+SA=E MD+Probiotics=F MD+LA=G

**S2 Table: SUCRA analysis: MeanRank table (PPD reduction on peri-implant mucositis)**

| treatment | SUCRA |
| --- | --- |
| A | 16.5 |
| B | 30.6 |
| C | 82.2 |
| E | 51.1 |
| F | 100.0 |
| G | 46.5 |
| H | 23.1 |

Label: MD=A MD+Laser=B MD+PBMT=C MD+SA=E MD+Probiotics=F MD+LA=G MD+APP=H

**S5 Fig: Inconsistency test** **(BoP reduction on peri-implant mucositis)**


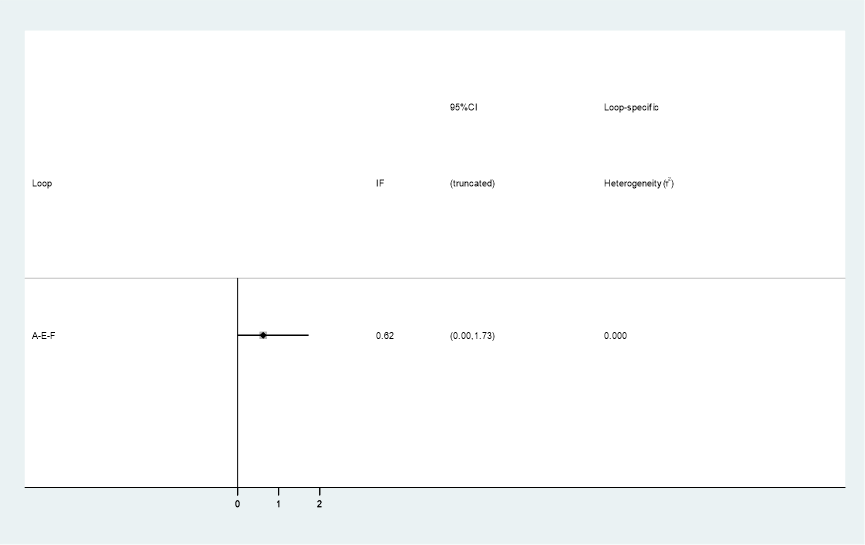


Label: MD=A MD+SA=E MD+Probiotics=F

**S6 Fig: Forest plot ((BoP reduction on peri-implant mucositis)**


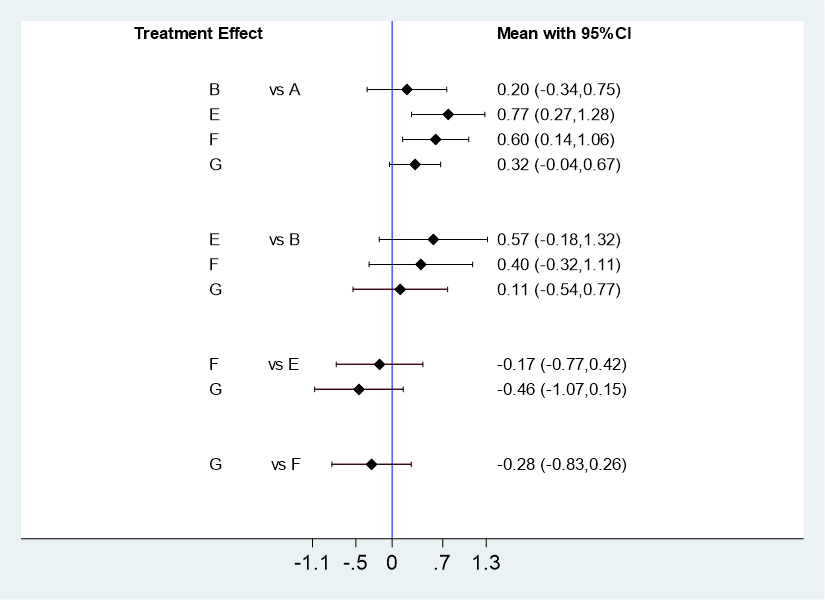


Label: MD=A MD+Laser=B MD+SA=E MD+Probiotics=F MD+LA=G

**S3 Table: SUCRA analysis: MeanRank table (BoP reduction on peri-implant mucositis)**

| treatment | SUCRA |
| --- | --- |
| A | 7.1 |
| B | 33.0 |
| E | 88.1 |
| F | 75.7 |
| G | 46.0 |

Label: MD=A MD+Laser=B MD+SA=E MD+Probiotics=F MD+LA=G

**S7 Fig: Inconsistency test (PLI reduction on peri-implant mucositis)
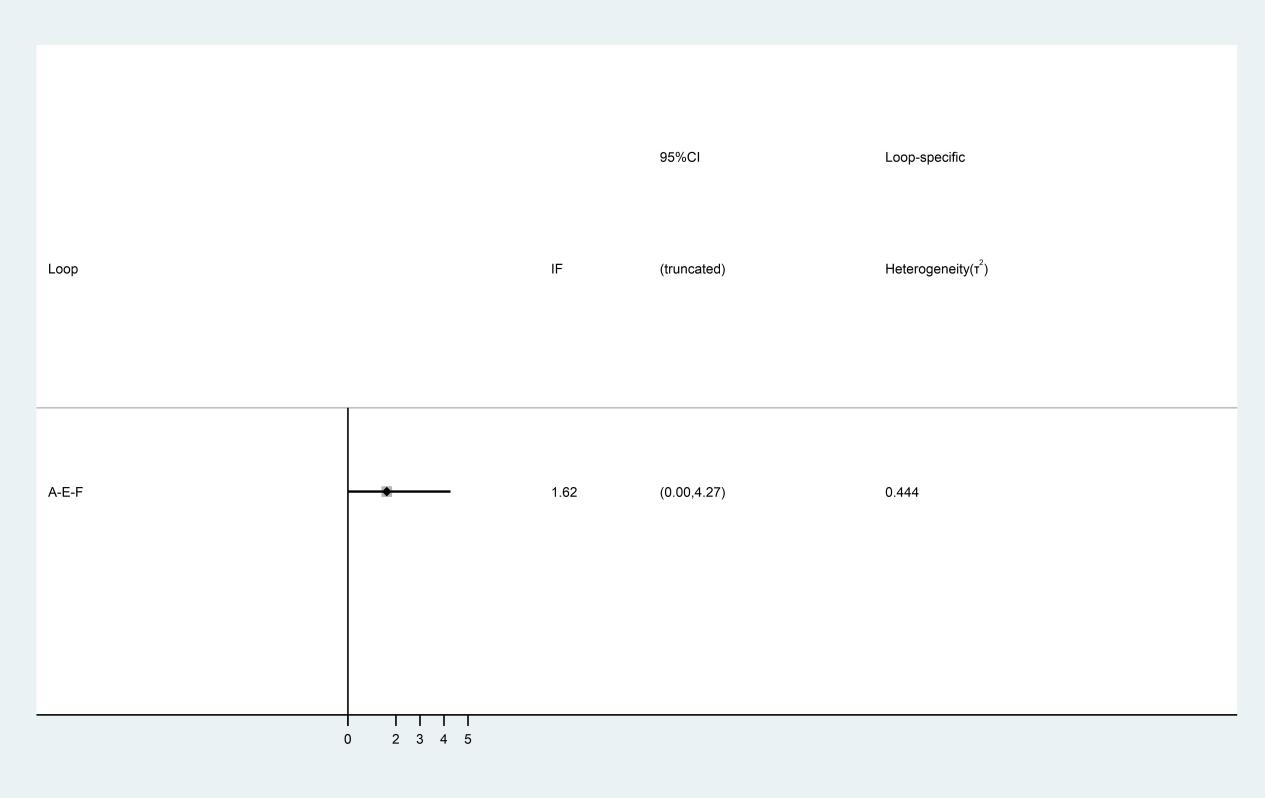
**

Label: MD=A MD+SA=E MD+Probiotics=F

**S8 Fig: Forest plot (PLI reduction on peri-implant mucositis)**


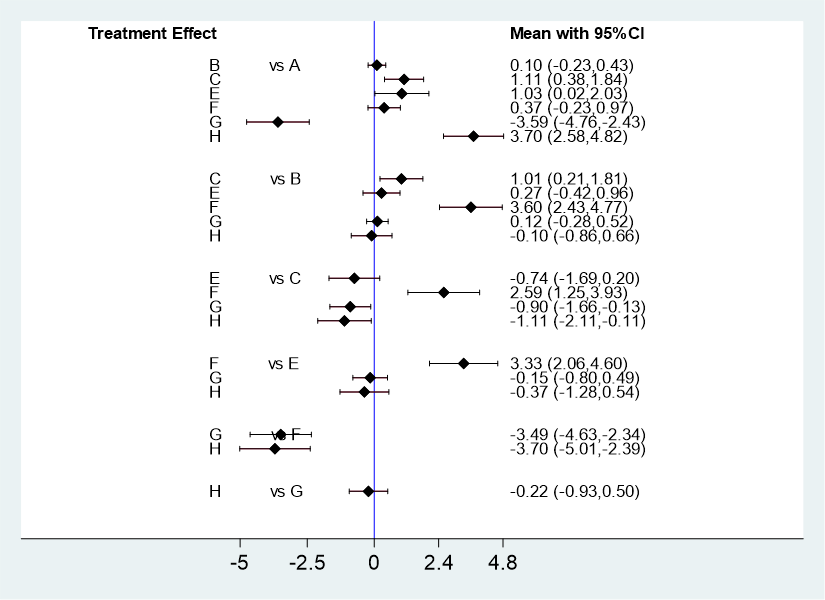


Label: MD=A MD+Laser=B MD+PBMT=C MD+SA=E MD+Probiotics=F MD+LA=G MD+APP=H

**S4 Table: SUCRA analysis: MeanRank table (PLI reduction on peri-implant mucositis)**

| treatment | SUCRA |
| --- | --- |
| A | 16.5 |
| B | 30.6 |
| C | 82.2 |
| E | 51.1 |
| F | 100.0 |
| G | 46.5 |
| H | 23.1 |

Label: MD=A MD+Laser=B MD+PBMT=C MD+SA=E MD+Probiotics=F MD+LA=G MD+APP=H

**S9 Fig: Forest plot (CAL changes on peri-implantitis)**


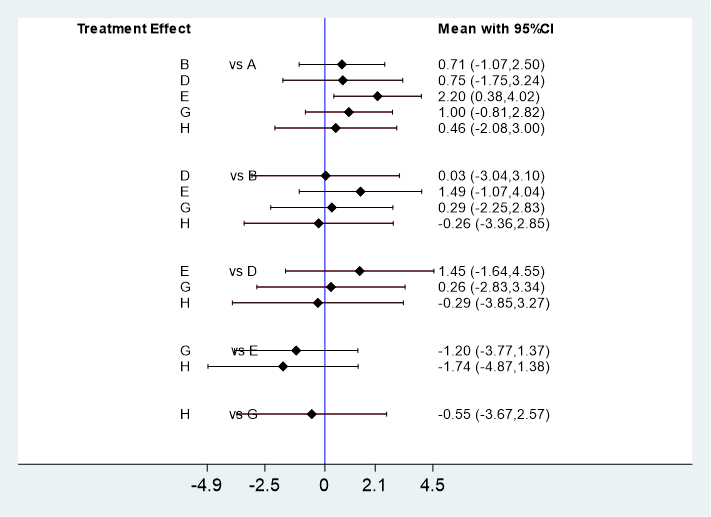
Label: MD=A MD+Laser=B MD+PDT=D MD+SA=E MD+LA=G MD+APP=H

**S5 Table: SUCRA analysis: MeanRank table (CAL changes on peri-implantitis)**

| treatment | SUCRA |
| --- | --- |
| A | 20.3 |
| B | 47.0 |
| D | 50.4 |
| E | 87.4 |
| G | 56.5 |
| H | 38.3 |

Label: MD=A MD+Laser=B MD+PDT=D MD+SA=E MD+LA=G MD+APP=H

**S10 Fig: Forest plot (MBL changes on peri-implantitis)**

Label: MD=A MD+Laser=B MD+SA=E MD+APP=H

**S6 Table: SUCRA analysis: MeanRank table (MBL changes on peri-implantitis)**

| treatment | SUCRA |
| --- | --- |
| A | 32.1 |
| B | 1.2 |
| E | 99.9 |
| H | 66.7 |

Label: MD=A MD+Laser=B MD+SA=E MD+APP=H
